# Surgical treatment of complex scapular fractures: a single centre prospective observational study

**DOI:** 10.1186/s13018-026-07247-8

**Published:** 2026-09-25

**Authors:** Sergio Ferraro, Andrea Pautasso, Riccardo Maria Lanzetti, Pipino Gennaro, Giai Via Alessio Davide Enrico, Fabio D’Angelo, Michele Francesco Surace, Filippo Migliorini, Nicola Maffulli

**Affiliations:** 1 Orthopaedic and Traumatology Unit, Sanremo Hospital, Via Giovanni Borea, 56, 18038 Sanremo, Italy; 2 Orthopaedic and Traumatology Department, ASST Sette Laghi – Circolo Hospital and Macchi Foundation in Varese - University Hospital, Viale Luigi Borri, 57, 21100 Varese, Italy; 3 Orthopaedic and Traumatology Unit, Department of Emergency and Acceptance, Azienda Ospedaliera San Camillo – Forlanin, Circonvallazione Gianicolense, 87, 00152 Rome, Italy; 4 Department of Orthopaedic and Trauma Surgery, Villa Erbosa Hospital, Gruppo San Donato, Via dell’Arcoveggio, 50, 40129 Bologna, Italy; 5 Department of Biotechnology and Life Sciences (DBSV), University of Studies Insubria, Via Ravasi 2, 21100 Varese, Italy; 6 Orthopaedic and Traumatology Department, Sant’Anna Hospital, Via Ravona, 20, San Fermo della Battaglia, Italy; 7 Department of Trauma and Reconstructive Surgery, University Hospital of Halle, Martin-Luther University Halle-Wittenberg, Ernst-Grube-Street 40, 06097 Halle (Saale), Germany; 8 Department of Clinical Orthopaedics, Eifelklink St Brigida, Simmerath, Germany Kammerbruchstraße 8, 52152; 9 Department of Life Sciences, Health, and Health Professions, Link Campus University, Via del Casale di San Pio V, 00165 Rome, Italy; 10 Orthopaedic and Traumatology Department, Sant’Andrea University Hospital, Via di Grottarossa 1035, 00189 Rome, Italy; 11 School of Medicine and Psychology, University of Rome “La Sapienza”, Piazzale Aldo Moro, 3/5, 00185 Rome, Italy

**Keywords:** Scapular fractures, Trauma, Scapular osteosynthesis, Scapular Judet approach

## Abstract

**Background:**

Conservative management is traditionally preferred for scapular fractures, but recent studies suggest that surgical intervention may improve outcomes in selected patients. This study evaluates functional and imaging outcomes, complication rates, and under-reported factors, such as duration of surgery and time from injury to definitive fixation, in surgically treated patients.

**Methods:**

This prospective observational study included 12 patients who underwent surgery for scapular fractures between 2016 and 2022. Inclusion criteria were complex scapular fractures, defined as displaced fractures with articular extension or floating shoulder (i.e., with associated ipsilateral clavicle fractures), and good psychophysical condition for postoperative evaluation. Exclusion criteria included isolated intra-articular glenoid fractures, associated proximal humerus fractures, and patients who did not complete the 1-year follow-up. Functional outcomes were assessed using the Quick-DASH, Constant-Murley Score, and Simple Shoulder Test, with imaging outcomes evaluated through follow-up imaging. Statistical analysis included t-test and linear regression.

**Results:**

The mean age at the time of injury was 48.3 ± 15.9 years. At one year, the Quick-DASH score was 13.6 ± 10.5%, the Constant-Murley Score improved to 87.9 ± 5.3, and the Simple Shoulder Test increased to 11.7 ± 0.50. Range of motion improved significantly across all parameters, especially between 6 and 12 months (*p* < 0.05), without statistically significant differences compared with the contralateral side at 1 year. A significant association between age and external rotation values was evident at six months.

**Conclusion:**

Open reduction and internal fixation (ORIF) of complex scapular fractures yield excellent functional recovery and high patient satisfaction. Accurate fracture classification and tailored surgical approaches optimise outcomes. These findings support the role of ORIF and emphasise the importance of the trauma-to-surgery interval and operative time.

## Introduction

Scapular fractures are relatively rare, accounting for 0.3–1% of all fractures, with an incidence of approximately 10 per 100,000 individuals per year [1]. Among these, fractures of the scapular body account for approximately 62% of cases, glenoid fractures 29%, and the remaining fractures involve the processes and scapular neck [1, 2].

These injuries occur predominantly in young males and are usually the result of high-energy trauma, particularly road traffic accidents and falls from height [3]. Given the high-energy mechanisms, scapular fractures are often associated with additional injuries, with a reported prevalence ranging from 35 to 98% [4]. Thoracic injuries, including rib fractures, lung contusions, pneumothorax, hemothorax, and sternal fractures, are commonly reported and contribute to an overall mortality rate of 10–15% [5–7].

The unique biological and mechanical properties of the scapula, including its fibro-muscular envelope and rich vascularisation, provide intrinsic stability and a low risk of fracture displacement [8]. Consequently, conservative management has historically been considered the standard treatment. However, several recent studies have demonstrated that operative treatment can provide excellent clinical and functional outcomes in selected patients when appropriate surgical indications are applied [9–12].

Long-term follow-up studies of conservatively treated scapular fractures have shown persistent symptoms and functional impairment in patients with residual deformity [13]. Likewise, surgical correction of symptomatic extra-articular scapular malunions has been associated with significant improvements in shoulder strength, range of motion, and Disability of the Arm, Shoulder, and Hand (DASH) scores [14].

Cole et al. [9] proposed six radiological criteria to guide surgical indication for scapular body fractures based on fracture displacement, glenoid alignment, articular involvement, and disruption of the superior shoulder suspensory complex. These criteria were adopted in the present study and are described in detail in the Materials and Methods section. The same authors [9] also proposed different surgical strategies according to fracture pattern, fracture comminution, and timing of surgery, including the Judet approach [15], the modified Judet approach [16], and minimally invasive plate osteosynthesis (MIPO) [17].

The aim of this single-centre prospective observational study is to evaluate functional and imaging outcomes, as well as complication rates, in patients with complex scapular fractures (defined as displaced fractures with articular extension or floating shoulder) and to compare these with those reported in the literature. Additionally, this study also aims to highlight under-reported factors, including surgical duration and the time from injury to definitive fixation.

## Materials and methods

### Study design and setting

An observational prospective study was conducted on patients who underwent surgical reduction and fixation for complex scapular fractures between January 1, 2016, and December 31, 2022, at the ASST Sette Laghi - University Hospital in Varese and Cittiglio (Italy).

### Inclusion and exclusion criteria

Inclusion criteria encompassed all patients with displaced fractures of the scapular body, associated with involvement of the articular component and/or the ipsilateral clavicle (floating shoulder), who were in good psychophysical condition for postoperative evaluation. Surgical indications had to meet the Cole criteria [9] based on computed tomography (CT) with multiplanar reconstructions (MPR) and 3D imaging and the following parameters: (1) Medial displacement of the articular surface relative to the lateral column fragment by at least 2 cm; (2) Glenoid-polar angle (GPA) less than or equal to 22°; (3) Fragment angulation greater than 45° in the sagittal view or angular deformity exceeding 30° with medialisation greater than 15 mm; (4) Glenoid surface fracture involving more than 25% of its area, with a fragment gap of at least 4 mm, and fracture lines extending into the scapular body; (5) Intra-articular step-off or gap of at least 4 mm; (6) Double disruption of the superior shoulder suspensory complex, particularly in cases of clavicular and scapular displacement of at least 10 mm or complete dislocation of the acromioclavicular joint associated with a scapular fracture displaced by more than 10 mm.

Polytrauma patients with neurological sequelae, psychiatric disorders, or limb amputations were excluded, as well as patients with isolated intra-articular glenoid fractures or associated proximal humerus fractures. Additionally, patients who did not complete both clinical and imaging follow-up assessments at 6 and 12 months were excluded from the study. Written informed consent was obtained from all patients included in the study for participation and for the use of their clinical data.

### Surgical technique and measures

All patients underwent surgery under general anaesthesia. Depending on the fracture pattern, patients were positioned either in a lateral decubitus or prone.

The most commonly used approach was the Judet approach, with an L-shaped skin incision following the scapular spine and the medial border of the scapula. The posterior portion of the deltoid was detached from the scapular spine, and the infraspinatus and teres minor muscles were elevated. This approach was employed in more comminuted fractures to allow better direct visualisation. In patients with less complex fractures (e.g., Ideberg type 2) or in younger patients, a modified Judet approach was preferred. This also involved an L-shaped skin incision, elevation of a deltoid flap, and the creation of operative windows: (1) between the scapular spine and the infraspinatus at its more medial portion, paying particular attention to avoid injury to the suprascapular nerve, allowing placement of the L-shaped plate on the medial aspect using a minimally invasive plate osteosynthesis (MIPO) technique without detaching the infraspinatus; and (2) between the infraspinatus and the teres minor. Ring fixation constructs were used, consisting of two dedicated plates: an L-shaped “boomerang” plate on the scapular body and a straight plate on the lateral column. In Ideberg type 2 fractures, only straight plates on the lateral column were used.

In patients with a floating shoulder, the ipsilateral clavicle fracture was addressed first through an anterior approach in the beach-chair position, to restore clavicular length before proceeding with scapular osteosynthesis. For both scapular and clavicular fractures, dedicated 3.5-mm Acumed^®^ LCP plates (5885 NW Cornelius Pass Road, Hillsboro, OR, USA) were used, with the option to apply either compression screws or locking screws. No drains were used; after accurate haemostasis, meticulous layered wound closure was performed to prevent hematoma formation.

Following surgery, patients were immobilised in a shoulder brace with the arm positioned at 15° of abduction and 15° of external rotation. The brace was maintained for 4 weeks during the daytime only and could be removed at night. Following suture removal, approximately 15 days postoperatively, gradual passive mobilisation of the shoulder was started, allowing up to 90° of forward flexion and abduction. Passive rotational movements were also permitted according to pain. After 30 days, structured physiotherapy was started, initially focusing on the recovery of the range of motion in all space planes, followed by progressive strengthening exercises. The study evaluated the time interval between trauma and definitive surgical treatment, intraoperative patient positioning, surgical duration, and the surgical approach performed.

### Outcome measures

Range of motion (ROM) in all directions and recovery of muscle strength (using a dynamometer – K Push^®^ Kinvent, Italy) were evaluated by the same surgeon (SF) in terms of clinical and functional outcomes during follow-up visits. Patients were evaluated at 6 months and at least 1-year post-surgery using the Quick-DASH disability questionnaire [18], the Constant-Murley Score [19], the Simple Shoulder Test (SST) [20], and the Visual Analogue Scale (VAS) [21]. Any complications encountered and their management were documented and analysed. Postoperative radiographic follow-up evaluations were conducted using at least two views (anterior-posterior and Y-View) and were scheduled at 1, 3, 6, and 12 months after surgery. Imaging assessments were performed to evaluate reduction quality and stability, potential hardware mobilisation, progression of fracture healing, and the presence of any morphostructural alterations (glenopolar angle (GPA) and glenoid medialization were measured at the final follow-up).

### Statistical analysis

The collected data were analysed using descriptive statistics and processed with IBM SPSS Statistics^→^ software. A paired-samples t-test was conducted to compare clinical outcomes at 6 months and 1-year post-surgery. A bivariate correlation analysis was then performed to assess relationships among variables, followed by linear regression for variables that showed high correlations. The threshold for statistical significance was set at *p* < 0.05.

## Results

### Patient characteristics

**Fig. 1:**
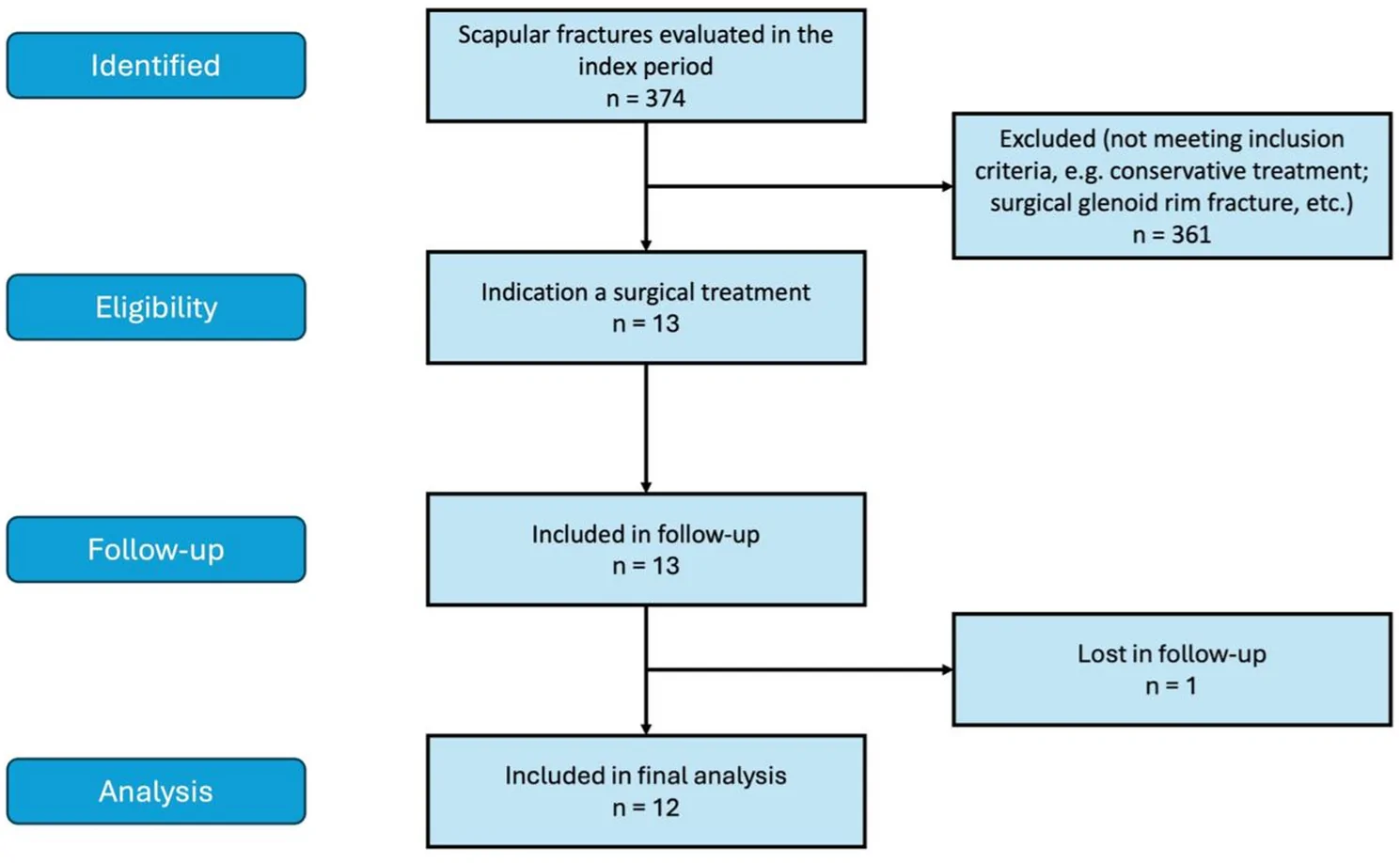
Flow diagram of patient inclusion and exclusion

During the study period, a total of 374 scapular fractures in 374 patients were evaluated at our institution; 13 patients underwent surgical reduction and fixation for scapular body fractures and met the inclusion criteria. One patient was lost to follow-up (Fig. 1).

The mean age of the 12 surgically treated patients included in the present study was 48.1 ± 14.3 years (range 26–73), and in 8 of 12 patients (66.7%) the non-dominant side was affected (Table 1).

**Table 1:** Baseline characteristics of the study population and surgery data

Variables		Data
Number of patients included		12
Age (years)		48.1 ± 14.3 (26–73)
Dominant side affected		4/12 (33.3%)
Cause of injury	Car accident	6/12
	Fall from height	4/12
	Pedestrian hit	2/12
Time from trauma to surgery (days)	9.6 ± 5.3 (4–23)	
Positioning	Prone	8
	Lateral	4
Surgical approach	Judet approach	8
	Modified Judet approach	4
Type of Surgery	2 plates with screws	8
	1 plate with screws	4
Mean Surgery Duration (minutes)	240.2 ± 92.7 (150–448)	

Fractures were classified by two experienced surgeons (SF and AP), with 100% interobserver agreement on fracture type classification. Three of the 12 fractures were purely extra-articular, without any involvement of the glenoid surface; all were associated with a clavicular fracture, consistent with a floating shoulder pattern. These extra-articular scapular fractures were classified according to the AO/OTA system [22, 23] as 14C0. Additionally, two patients presented a simple 14B1 pattern, and one presented a comminuted 14B2 scapular body fracture. The remaining nine fractures were classified according to the modified Ideberg classification [24, 25] as follows: two type 2 fractures, five type 5a fractures, and two type 5c fractures. One of these patients also presented with a floating shoulder pattern.

**Fig. 2:**
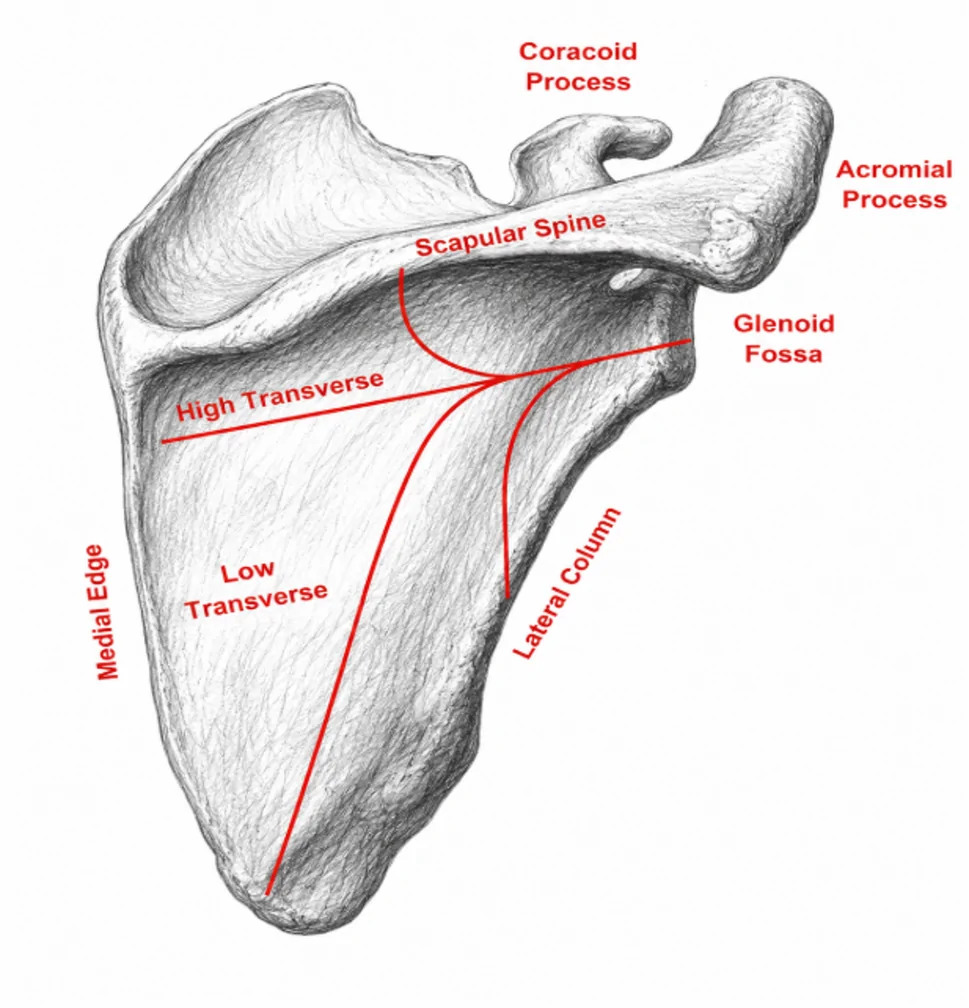
Anatomical fracture patterns of the scapula observed in the study population. Schematic illustration showing the main fracture lines: lateral column, scapular spine, coracoid base, high transverse, and low transverse patterns

In terms of anatomical fracture patterns (Fig. 2), 11 patients presented with a lateral column fracture, one patient had also a fracture at the base of the coracoid process, and nine patients showed a transverse scapular body fracture. Three of these patients exhibited a comminuted pattern, involving both high and low transverse fracture lines (Table 2).

**Table 2:** Distribution of scapular fractures. (AO/OTA: Arbeitsgemeinschaft für Osteosynthese/Orthopaedic Trauma Association)

Fracture type		Number
Involving Intra-Articular Portion		9/12
Ideberg Classification	Type 2	2/9
	Type 5a	5/9
	Type 5c	2/9
Involving Extra-Articular Portion Only		3/12
AO/OTA Classification	14C0 + 14B1	2/3
	14C0 + 14B2	1/3
Anatomical Classification	Glenoid Fracture	9/12
	Simple	7/9
	Comminuted	2/9
	Lateral Column Fracture	11/12
	Transverse Scapular Body Fracture	9/12
	Base of Coracoid Fracture	1/12
Floating Shoulder		4/12

All fractures resulted from high-energy trauma in the context of polytrauma (car accident: 6/12; fall from height: 4/12; and pedestrian hit: 2/12). The mean time interval between the traumatic event and surgical intervention was 9.6 ± 5.3 days (range 4 − 23).

### Surgical data

**Fig. 3:**
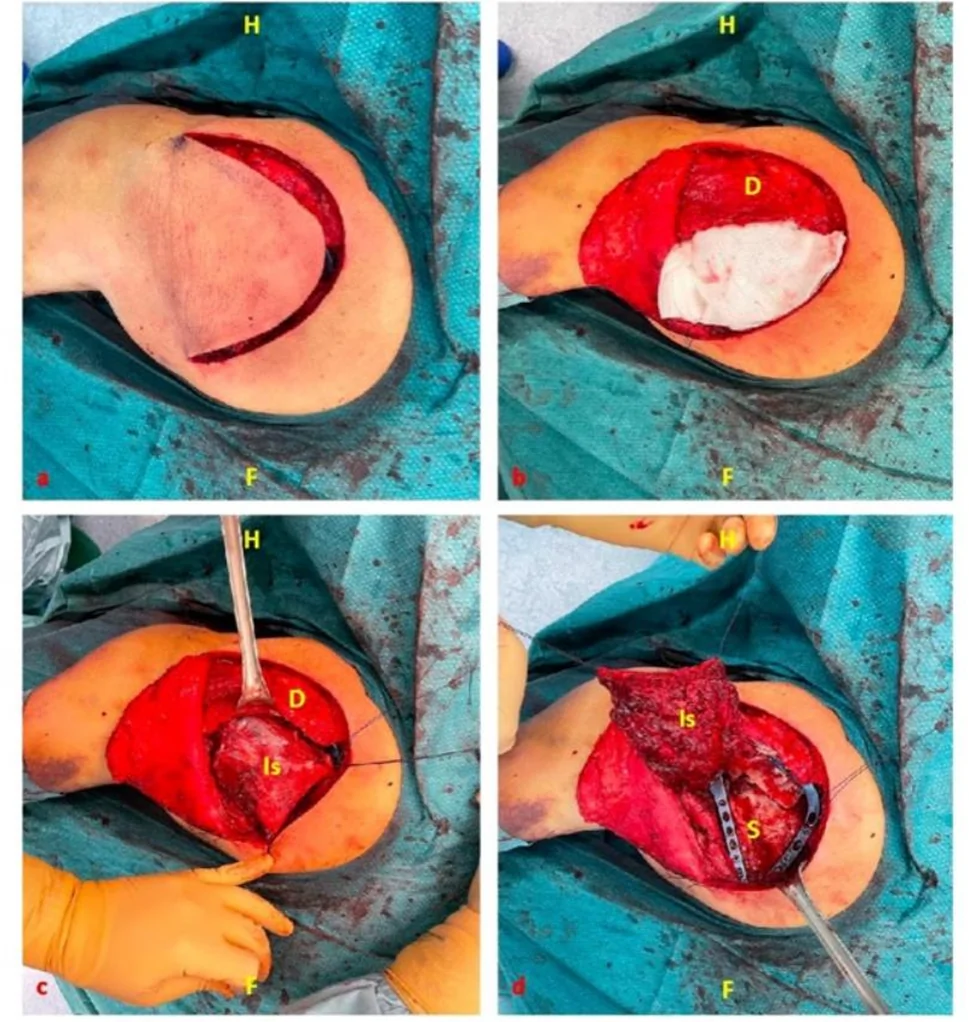
Intraoperative images of the extended Judet approach. **a** Skin incision. **b** Exposure of the deltoid (D). **c** Identification and detachment of the infraspinatus (Is). **d** Reflection of the infraspinatus and exposure of the scapula (S) for fixation. H, head; F, foot

Regarding surgical treatment, eight patients were positioned prone, while the remaining four patients were positioned in lateral decubitus. In four of 12 patients, a second-stage surgical procedure in the beach-chair position was required to undertake an anterior approach. In eight of 12 patients, an extended Judet approach was performed, involving medial detachment and reflection of the infraspinatus muscle (Fig. 3). Four patients were treated with a modified Judet approach.

In eight patients, two pre-contoured 3.5 mm Acumed^®^ LCP plates were used. In the remaining four patients, a single 3.5 mm Acumed^®^ LCP plate was applied to the lateral column. The mean surgical duration was 240.2 ± 92.7 min (range 150–448 min).

### Clinical outcomes

#### Functional scores

The mean Constant-Murley Score was 72 ± 9.4 (range 52–83) at six months, improving to 87.9 ± 5.3 (range 77–96) at one year. The Simple Shoulder Test (SST) score followed a similar trend, with a mean of 9.6 ± 2.1 (range 7–12) at six months and 11.7 ± 0.5 (range 11–12) at twelve months, with eight patients reaching the maximum score. The final Quick-DASH disability score at one year averaged 13.6 ± 10.5% (range 2.3–31.8%). By six months post-trauma, all patients had returned to work, although eight required modified duties due to functional limitations. After one year, all patients were fully capable of performing their occupational tasks. Statistically significant differences (*p* < 0.05) were observed between the 6- and 12-month data for all scores (CMS, ROM, SST, and VAS). No statistically significant differences were evident comparing the ROM of the operated shoulder with the contralateral side at 1 year of follow-up. Associated injuries such as rib fractures, pneumothorax, or lung contusions did not influence functional recovery or outcome scores at follow-up.

#### Range of motion (ROM)

ROM at six months showed a mean forward flexion (FF) of 148.8 ± 16.3 degrees (range 120–180), abduction (AB) of 127.5 ± 34.1 (range 60–180), and external rotation (ER) of 48.3 ± 15.9 (range 20–80). Mean internal rotation (IR) reached the sacroiliac (SI) joint. Specifically, one patient reached T12, three reached the waist, three reached the buttocks, three reached the iliac crest, and two reached the thigh. By 1 year, ROM had significantly improved, reaching a mean of 165 ± 9 degrees for forward flexion (range 150–180), 152.5 ± 22.9 degrees for abduction (range 105–180), and 65 ± 12.4 degrees for external rotation (range 40–80). Internal rotation improved to a mean level of T12, with six patients reaching the interscapular region, three reaching T12, and three reaching the waist.

When stratifying the population by age, 5 patients (41.7%) were under 40 years old at the time of trauma, while 12 (58.3%) were over 40. The younger group (< 40 years) demonstrated a significantly faster recovery in joint mobility. By six months, forward flexion, abduction, and external rotation had already reached 94.7%, 89.5%, and 78.9%, respectively, relative to pre-trauma maximum ROM (set as the ROM of the contralateral side), with full recovery achieved by one year. In contrast, internal rotation recovery remained lower, with a mean recovery rate of 81.8% at 12 months. In patients over 40 years of age, internal rotation recovery followed a similar pattern to that of the younger group.

**Fig. 4:**
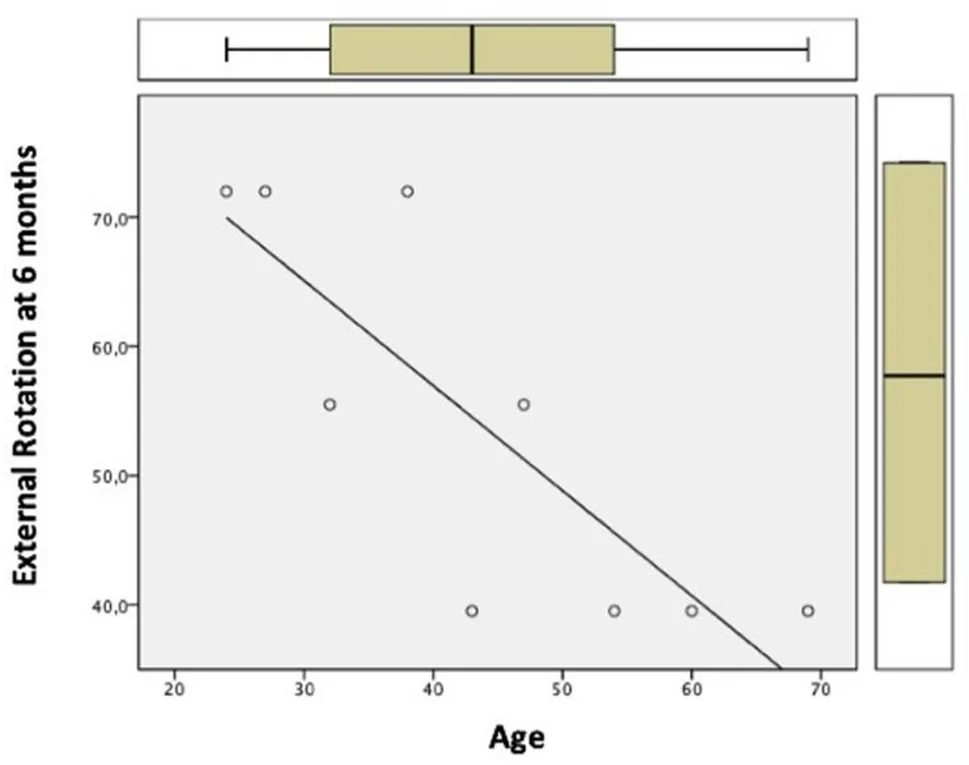
Correlation between age and external rotation at 6 months

A bivariate correlation analysis of age and ROM parameters (FF, AB, IR, and ER) revealed a statistically significant association between age (less than 40 years) and external rotation at 6 months, with R^2^ = 0.677 and *p* < 0.05. Linear regression analysis confirmed this association, as illustrated in Fig. 4.

### Imaging outcomes

The mean consolidation time with evident bone healing at follow-up was 2.8 ± 0.8 months (range 2–4 months). No patients developed malunion, delayed union, nonunion, or glenoid medialization. The GPA was fully restored to the normal range, with a mean of 40.8 ± 2.9 (36–45).

**Table 3:** Summary of functional outcomes and clinical scores. (SI: sacrum-iliac.)

Clinical outcomes/variables		Data	
		6 months	1 year
Constant-Murley Score		72 ± 9.4 (52–83)	87.9 ± 5.3 (77–96)
Range of Motion	Forward Flexion	148.8 ± 16.3 (120–180)	165 ± 9 (150–180)
	Abduction	127.5 ± 34.1 (60–180)	152.5 ± 22.9 (105–180)
	External Rotation	46.7 ± 15.6 (20–80)	65 ± 12.4 (40–80)
	Internal Rotation	SI Joint (Thigh – Waist)	Waist (SI Joint – Interscapular region)
Simple Shoulder Test		9.6 ± 2.1 (7–12)	11.7 ± 0.5 (11–12)
Visual Analogue Scale		1.0 ± 1.5 (0–4)	0.1 ± 0.3 (0–1)
Quick-Dash (%)			13.6 ± 10.5 (2.3–31.8)
Consolidation Time (months)		2.8 ± 0.8 (2–4)	
Glenopolar angle post-surgery (degrees)		40.8 ± 2.9 (36–45)	
Surgical complications		4	

### Complications

One patient experienced a left axillary nerve deficit, presenting with hypoesthesia in the area of innervation and deltoid muscle weakness. He was a 66-year-old man who sustained an Ideberg type 5c scapular fracture following a car accident and was treated surgically through a Judet approach with dual plate fixation. Two patients developed suprascapular nerve neurapraxia, with weakness of the infraspinatus muscle: these patients sustained polytrauma following a pedestrian accident and a fall from height, respectively, resulting in a floating shoulder associated with an Ideberg type 5c scapular fracture in one case and a 14C0 + 14B1 extra-articular fracture in the other. Both patients were treated surgically through a Judet approach with double-ring plate fixation. The three patients who developed these symptoms during the first months of follow-up underwent electromyography, which confirmed the deficit. However, at the 6-month evaluation, all demonstrated complete recovery. Additionally, one patient developed a seroma as an early postoperative complication; this was successfully drained without further issues.

## Discussion

Over the past two decades, the approach to scapular body fractures has evolved, with an increasing trend toward surgery. However, no consensus has been reached regarding surgical indications [12, 23, 26–31]. The aim of this single-centre prospective observational study was to evaluate functional and imaging outcomes and complication rates in patients with complex scapular fractures and to compare these with those reported in the literature. Additionally, this study aimed to highlight under-reported factors, including duration of surgery and the time from injury to definitive fixation. In our study, final follow-up ROM values were 165 ± 9° (range 150–180°) for forward flexion, 152.5 ± 22.9° (range 105–180°) for abduction, and 65 ± 12.4° (range 40–80°) for external rotation, with a mean internal rotation reaching the waist. Notably, all functional scores and ROM parameters improved significantly between the 6- and 12-month follow-up evaluations, highlighting that recovery after ORIF is gradual and should be supported by structured postoperative physiotherapy.

Cole et al. [9] analysed 61 patients with a mean follow-up of 33 months (range 12–138). At final follow-up, the mean ROM was 154° (range 60°–188°) for forward flexion, 106° (range 70°–160°) for abduction, and 66° (range 18°–103°) for external rotation. Notably, the mean ROM of the uninjured contralateral shoulder was 159° (range 126°–188°) for flexion, 108° (range 78°–160°) for abduction, and 70° (range 18°–106°) for external rotation. The mean and median DASH scores reported by Cole et al. [9] at final follow-up were 12.1% and 4%, respectively (range 0–54; mean: 10.1%). Additionally, SF-36 scores were comparable to those of the general population. Of their 49 patients, 36 (73%) returned to work and pre-injury activities, while six (12%) were unable to resume their occupations because of non-scapular-related injuries. Similarly, in the present study, patients at final follow-up had a mean Quick-DASH score of 13.6 ± 10.5% (range 2.3–31.8%) and a mean Constant-Murley Score of 87.9 ± 5.3 (range 77–96).

Michelitsch et al. [11], following the surgical indications proposed by Cole et al. [9], analysed a cohort of 36 patients with scapular body fractures, with a mean follow-up of 33 months (range 8–70). Functional outcomes were excellent, with a mean Quick-DASH score of 0% and a mean Subjective Shoulder Value (SSV) of 95 (range 90–98). The median Numerical Rating Scale (NRS) for pain was 0 (range 0–1), and the median Activity of Daily Living (ADL) score was 1 (rang: 1–1). Radiographic fracture healing was achieved in all patients, with no failures of internal fixation or malunion. However, two patients (7.7%) required early revision surgery, one patient (3.8%) developed frozen shoulder three months postoperatively, and another (3.8%) experienced a superficial wound infection.

In our study, a 100% union rate was achieved, with a mean time to union of 2.8 ± 0.8 months (range 2–4). No complications such as malunion, delayed union, nonunion, or glenoid medialization were detected. The GPA was restored to within the normal range, with a mean value of 40.8 ± 2.9 (36–45). This reinforces the trend for surgical management of selected fractures. Indeed, malunions following conservatively treated scapular body fractures can lead to significant disability [14]. In a retrospective series, five patients with malunions involving the scapular body and neck underwent surgical treatment to restore the normal anatomy through osteotomies and reorientation of the scapular segments. Despite the technically demanding and highly invasive nature of these procedures, all patients experienced radiographic healing at a mean follow-up of 39 months (range 18–101), reported no residual shoulder pain, and were satisfied with their surgical outcomes. Four of five patients returned to their pre-injury occupations and activities. The Quick-DASH score improved from a preoperative mean of 39% (range 27–58) to a postoperative mean of 10% (range 0–35), with no reported complications.

The same research group [32] conducted a retrospective analysis on 84 patients who underwent surgery for scapular body fractures. By strictly adhering to the criteria described by Cole and colleagues, they reported a 100% fracture healing rate, with three patients developing malunion and no surgical complications.

Although favourable surgical outcomes have been reported in the literature when appropriate indications are followed, the volume of these procedures performed per centre remains low. These fractures often occur in polytrauma patients who require initial admission to the Intensive Care Unit, delaying surgery until general stabilisation is achieved. In the present study, the mean time from trauma to surgery was 9.6 ± 5.3 days (range 4.0–23.0).

In the present study, the mean operating room time was 240.2 ± 92.7 min (range 150.0–448.0). These data reflect both the limited number of patients and the complexity of the fractures included, three of which were floating shoulders requiring initial clavicle fixation. Such prolonged surgical time may increase the risk of complications, including surgical site infection, systemic infection, anaesthetic-related complications, and blood loss. Finally, the surgical procedure remains technically demanding in fracture reduction and fixation. Although these factors may discourage surgical treatment in borderline cases, conservative management should remain reserved for minimally displaced fractures that do not meet established surgical indications. In contrast, patients fulfilling accepted radiological criteria for surgery are likely to benefit from anatomical reduction and stable fixation despite the technical complexity of the procedure [7, 33].

Regarding the surgical technique, the extended posterior Judet approach was performed more frequently. This approach, which is invasive and requires longer operative times, allows direct visualisation of the fracture from the scapular spine base to the lower third of the body and laterally to the posterior half of the glenoid (Fig. 3), facilitating precise reduction and fixation. However, it also presents potential disadvantages. The prolonged retraction of the infraspinatus muscle and extensive surgical exposure may increase the risk of postoperative complications and may negatively affect rehabilitation, potentially leading to external rotation weakness. The data of this study demonstrated a significant correlation between age and external rotation values at six months (R^2^ = 0.677; *p* < 0.05), with linear regression analysis confirming this association (Fig. 4).

In our series, one patient developed a transient axillary nerve deficit, and two patients experienced suprascapular nerve neurapraxia, resulting in infraspinatus muscle weakness. All three patients recovered completely within six months. Because these patients sustained high-energy polytrauma, it was not possible to determine whether the neurological deficits were caused by the initial traumatic event or by surgical exposure. Previous studies have reported that brachial plexus and peripheral nerve injuries are not uncommon following high-energy scapular fractures and shoulder girdle trauma, particularly in patients with floating shoulder or severe thoracic trauma, with most cases representing neurapraxia and showing spontaneous recovery during follow-up [34]. Although the extended Judet approach requires extensive soft-tissue dissection, careful surgical technique and meticulous handling of the suprascapular and axillary nerve regions can minimise iatrogenic complications, which remain uncommon [35].

Finally, most scapular body fractures identified intraoperatively do not fit within these existing classification systems [30]. This discrepancy arises because most classifications were developed using conventional radiographs or 2D CT scans, which may not fully capture the complexity of fractures. Today, 3D CT reconstructions represent the gold standard for accurate diagnosis to assess medialization of the lateral edge, glenoid-polar angle, and scapular angulation [30]. A classification distinguishing between high and low transverse scapular body fractures has important clinical implications. In low transverse fractures, the lateral border of the proximal fragment is sufficiently long to allow plate fixation with two screws, making surgery feasible via a modified or minimally invasive Judet approach. Conversely, high transverse fractures require a plate extending to the posterior glenoid surface or a reconstruction plate contoured along the posterior scapular neck, necessitating a more extensive approach. Bartoníček et al. [30] also emphasised the role of the lateral edge of the scapula as a crucial element for reduction and internal fixation; yet, no classification system specifically addresses fractures of this region. Stable lateral border fixation is the key step to achieve anatomic reconstruction, while medial edge fixation primarily serves to neutralise shear and rotational forces [36].

Our findings support the role of open reduction and internal fixation (ORIF) in achieving good functional outcomes and high patient satisfaction in the management of complex scapular fractures. Stable fracture fixation enables early mobilisation, preventing delays in functional recovery from prolonged immobilisation. Furthermore, restoring proper articular congruency reduces the risk of degenerative joint disease.

This study has several limitations. The small sample size (potential selection bias) and the absence of a direct comparison between conservative (control group) and surgical treatments prevent definitive conclusions regarding the superiority of one approach over the other. Furthermore, regarding the observed neurological deficits, as the patients were intubated at the scene and upon arrival at the Emergency Department, it was not possible to determine whether these deficits resulted from surgery or were pre-existing.

However, the strength of this study lies in the detailed classification of scapular body fractures for each patient, combined with a comprehensive assessment of functional and imaging outcomes.

## Conclusion

Conservative treatment remains widely employed for scapular fractures. Surgical management is guided by the principle that deformity and impaired function are closely correlated. Restoring scapular length, alignment, and rotation can significantly improve clinical outcomes.

Accurate diagnosis requires high-quality radiographs and 3D CT scans to assess key parameters, including medialization, glenoid-polar angle, and scapular angulation. Extended posterior approaches, particularly the Judet approach, offer excellent visualisation and are preferable in complex cases or when surgery is delayed. Lateral column reduction remains a crucial surgical objective, followed by correction of the medial border alignment as needed.

## Data Availability

The datasets used and/or analysed during the current study are available from the corresponding author on reasonable request.

## Abbreviations

M:F: Male:Female
DASH: Disability of the Arm, Shoulder, and Hand
ORIF: Open reduction and internal fixation
MIPO: Minimally Invasive Plate Osteosynthesis
CT: Computed tomography
MPR: Multiplanar reconstructions
3D: Three dimensional
GPA: Glenoid-Polar Angle
SST: Simple Shoulder Test
VAS: Visual Analogue Scale
AO/OTA: Arbeitsgemeinschaft für Osteosynthese/Orthopaedic Trauma Association
LCP: Locking Compression Plate
ROM: Range of motion
FF: Forward flexion
AB: Abduction
ER: External rotation
IR: Internal rotation
SSV: Subjective Shoulder Value
NRS: Numerical Rating Scale
ADL: Activity of Daily Living
